# Outcomes of Endocarditis in Pregnancy: A Single-Center Experience

**DOI:** 10.1093/ofid/ofad470

**Published:** 2023-09-20

**Authors:** Kayle Shapero, Sami El-Dalati, Kathryn Berlacher, Christina Megli

**Affiliations:** Heart and Vascular Institute, University of Pittsburgh Medical Center, Pittsburgh, Pennsylvania, USA; Department of Infectious Disease, University of Kentucky Medical Center, Lexington, Kentucky, USA; Heart and Vascular Institute, University of Pittsburgh Medical Center, Pittsburgh, Pennsylvania, USA; Department of Obstetrics, Gynecology and Reproductive Sciences University of Pittsburgh School of Medicine, Magee Women's Research Institute, Pittsburgh, Pennsylvania, USA

**Keywords:** infective endocarditis, maternal mortality and morbidity, opioid use disorder, pregnancy

## Abstract

**Background:**

The incidence of infective endocarditis (IE) in pregnancy is rare (0.006%), with increasing prevalence during the opioid epidemic. IE in pregnancy is associated with high rates of mortality and morbidity, and existing data on outcomes in pregnancy are limited. Our study compares the outcomes of pregnant patients with IE with those of nonpregnant patients.

**Methods:**

Patients diagnosed with IE during pregnancy and 30 days postpartum between 2014 and 2021 were identified by International Classification of Diseases, Clinical Modification, Ninth and Tenth Edition codes. Pregnant cases were matched to nonpregnant reproductive-age endocarditis patients in a 1:4 ratio. Data were collected and validated through chart review.

**Results:**

One hundred eighty patients with IE were identified; 34 were pregnant or within 30 days postpartum at diagnosis. There were higher rates of hepatitis C and opioid maintenance therapy in the pregnant patients. The etiology of IE in pregnant patients was predominantly *S. aureus* (methicillin-resistant/sensitive *S. aureus*), whereas nonpregnant woman had greater microbiological variation. We observed comparable rates of valve replacement (32.4% vs 29%; *P* = .84) and 2-year mortality (20.6% vs 17.8%; *P* > .99) in pregnant patients. There were nonsignificantly higher rates of pulmonary emboli (17.6% vs 7.5%; *P* = .098) and arrhythmia (17.6% vs 9.6%; *P* = .222) among pregnant patients. There were high rates of intravenous drug use relapse in both groups (>40%).

**Conclusions:**

We observed similar rates of mortality in the pregnant IE patients. We observed a microbial predilection for *S. aureus* in pregnancy, suggesting that the pregnancy physiology may select for this microbiologic etiology. This study, which represents the largest single-center retrospective review of IE in pregnancy, suggests that surgical intervention may be performed safely in the postpartum period.

Cardiovascular disease and infection are the leading causes of maternal mortality and severe morbidity, with infective endocarditis (IE) in pregnancy representing the intersection of these diseases. While the incidence of IE in pregnancy has historically been reported as rare (0.006%) [1], increasing incidence rates in pregnant and nonpregnant patients have been reported, paralleling the ongoing opioid epidemic [2, 3]. Persons who inject drugs are at increased risk for severe bacterial infections leading to IE, and the majority of cases of IE in pregnancy are now attributable to opioid use disorder [2, 3]. The physiologic and immune adaptations to pregnancy may increase the risk for complications and adverse outcomes. Additionally, the consideration of both maternal and fetal well-being complicates care [4]. Existing data on outcomes of IE in pregnancy are primarily limited to case reports and case series, and there are no clear recommendations regarding optimal management.

Cardiovascular adaptations to pregnancy confer a substantial risk of hemodynamic compromise due to valvular disease during pregnancy and in the postpartum period. Exact mortality data for IE in pregnancy are unknown, with reports ranging from 5% to 33% [1, 2]. Recent studies demonstrate improving outcomes, likely due to early diagnosis and treatment, which range from medical management to surgical intervention depending on the severity of disease and infectious complications [1, 3]. For those patients with significant hemodynamic compromise prompting consideration of extracorporeal membrane oxygenation (ECMO), there has been recent limited evidence that ECMO does not confer excess maternal risk in pregnancy [5]. While maternal outcomes from cardiac surgery are *reportedly* similar to those of nonpregnant female patients, the current literature is limited to case series and retrospective reviews. The use of cardiopulmonary bypass and cardiac surgery in pregnancy is still controversial, and data on rates of surgical intervention are limited [2]. Management decisions regarding timing of valve surgery are complex given poor fetal outcomes reported with cardiopulmonary bypass [6].

Given the lack of current data, we performed a retrospective single-center cohort study of IE outcomes in pregnant patients compared with nonpregnant counterparts to assess the impact of pregnancy on IE outcomes. Our study defines the outcomes of pregnant and nonpregnant patients with IE and identifies antenatal characteristics associated with maternal and neonatal morbidity and mortality.

## METHODS

### Patient Population

The search criteria for this study were limited to hospital admissions that occurred between 2014 and 2021 at the primary obstetric hospital in a large health care system. These dates ensured the use of both the ninth and tenth editions of the International Classification of Diseases, Clinical Modifications (ICD-CM-9/10). Patients were included in the study if they were of female sex, were of reproductive age (18–45), had at least 1 ICD code indicating IE, and had an encounter code for an echocardiogram within 6 months of IE diagnosis (Supplementary Table 1). For patients with multiple IE admissions, the first index admission within the system during that time frame was used. We included patients who were diagnosed with definite or possible IE according to the modified Duke's criteria even if no echocardiographic evidence of endocarditis was identified [7]. Within this cohort of patients, chart review was performed to identify those who were pregnant or within 30 days postpartum at the time of IE diagnosis. This yielded 2 cohorts of reproductive-age endocarditis patients: pregnant and nonpregnant. Patient data were validated through chart review of a sampling of nonpregnant female patients with IE of reproductive age in a 1:4 ratio in order to control for rate events not related to pregnancy that could impact outcomes. Of note, patients were included in the pregnant category if they were diagnosed with IE up to 30 days postpartum. This time frame was selected as the hormonal and physiologic changes of pregnancy can persist for up to 6 weeks postpartum. A flowchart describing the study design is included as Supplementary Figure 1.

Primary outcomes included mortality (in-hospital, 1-year, and 2-year) and valve replacement/repair. Secondary outcome included other valve surgeries, a thromboembolic event, arrhythmia, intubation, use of mechanical support, use of extracorporeal membrane oxygenation, intensive care unit (ICU) admission, and length of ICU and hospital stay. Primary and secondary outcomes are detailed in Supplementary Table 2.

### Definitions

Diagnosis of hepatitis C was based on chart review and included patients with prior positive hepatitis C antibody or viral load. Leukocytosis/leukopenia in pregnancy was defined by the reference values during each trimester of pregnancy (first trimester 5.7–13.6 × 10^9^/L, second trimester 5.6–14.8 × 10^9^/L, third trimester 5.9–16.9 × 10^9^/L) [8]. Persistent infection was defined as persistent bacteremia or sepsis despite adequate antibiotic therapy for >5–7 days [9]. Pulmonary embolus was defined as an embolus causing occlusion of the pulmonary artery, distinct from septic pulmonary nodules. Surgical indications were defined by the 2014 American College of Cardiology/American Heart Association guidelines on the Management of Patients with Valvular Heart Disease [9]. A list of outcomes can be found in Supplementary Table 2. Neonatal outcomes were calculated using the denominator of patients who remained pregnant until at least 23 weeks’ gestational age with a viable intrauterine pregnancy at that time.

### Statistical Analysis

Demographic, clinical, and maternal outcomes–related variables were compared. Statistical analysis was done using STATA (StataCorp LLC, College Station, TX, USA). Categorical variables were analyzed using Fisher exact *t* tests. Continuous variables were compared using a Fisher exact *t* test or Mann-Whitney *U* test (if data did not follow a normal distribution). A Cox regression hazard ratio was not utilized as we were not assessing time to event, but rather dichotomous data/events. This study was approved via the University of Pittsburgh Medical Center Internal Review Board (STUDY21060177).

## RESULTS

### Population Characteristics

Two hundred twenty-three total patients were identified with ICD codes for endocarditis and procedural codes for echocardiograms within 6 months of diagnosis. Forty-three total patients were excluded either because they did not meet definitive or possible IE or because their admission for IE occurred outside of the UPMC health care system. This resulted in 180 total IE patients, 34 of whom were pregnant or recently postpartum, and 146 control patients (Supplementary Figure 1). The majority of pregnancy-associated IE occurred during the index admission for delivery (76.5%), and the remainder occurred postpartum. Demographics are described in Table 1. Pregnant patients with IE were significantly younger than nonpregnant patients (29.6 years vs 32.6 years; *P* = .01). There were more Caucasian patients in the pregnant cohort (97% vs 85.6%; *P* = .08). There were also higher rates of hepatitis C (68% vs 42%; *P* = .01) in pregnant patients. There were lower rates of hypertension, hyperlipidemia, coronary artery disease, and diabetes among pregnant patients, although these differences were not statistically significant. Rates of mental health disorders were high in both groups, 44% in pregnant patients and 36% in nonpregnant (*P* = .44). There were higher rates of incarceration in pregnant as compared with nonpregnant patients (8.8% vs 5.7%; *P* = .08), although this was not statistically significant. In terms of IE risk factors, there were similar rates of congenital heart disease (11.8% vs 7.5%; *P* = .49), bicuspid aortic valve (5.8% vs 4.1%; *P* = .60), history of IE (17.7% vs 27.4%; *P* = .28), prosthetic valves (2.9% vs 10%; *P* = .34), and intravenous drug use (85.3% vs 80.8%; *P* = .63). Treatment with medication for opioid use disorder (58.5% vs 24%; *P* ≤ .001) was higher among pregnant patients.

**Table 1.** Demographics

	Nonpregnant Average		Pregnant Average		*P* Value
	Median or Count	Percentage or IQR/SD	Median or Count	Percentage or IQR/SD	
Demographics	…	…		…	…
Age at delivery, y	32.6	6.9	29.6	5.8	.011
Race	…	…		…	…
White	125	85.6	33	97.1	.082
Black	12	8.2	1	2.9	.467
American Indian	1	0.7	0	0	>.99
Unspecified/declined	10	6.9	0	0	.212
Medicaid/Medicare	105	71	26	76.5	.673
Comorbidities	…	…		…	…
HTN	23	15.8	3	8.8	.42
HLD	6	4.1	0	0	.596
CAD	1	0.7	0	0	>.99
CKD	10	6.6	0	0	.212
Obesity	8	5.5	1	2.9	>.99
CVA/DVT	14	9.6	3	8.8	>.99
Hepatitis C	62	42.5	23	67.6	.012
DM	17	11.6	1	2.9	.203
Thyroid disease	3	2.1	1	2.9	.571
Asthma	16	11.0	3	8.8	>.99
Smoking history	102	69.9	26	76.4	.532
IVDU	118	80.8	31	91.2	.208
Mental health d/o	53	36.3	15	44.1	.435
Incarceration	3	5.7	3	8.8	.082
Endocarditis risk factors	…	…		…	…
Native valve predisposition: bicuspid Aortic valve	6	4.1	2	5.8	.596
Prosthetic valve	19	13.0	2	5.9	.375
Intracardiac devices (ICD/defib)	2	1.4	0	0	>.99
Congenital heart disease	11	7.5	4	11.8	.49
History of endocarditis	40	27.4	6	17.7	.28
IVDU	118	80.8	29	85.3	.63
Chronic IV access	6	4.1	1	2.9	>.99
Protective risk factors:	…	…		…	…
Medication for opioid use disorder	35	24.0	20	58.8	<.001
Methadone	11	7.5	7	20.6	.05
Suboxone	24	16.4	13	38.2	.008

Abbreviations: CAD, coronary artery disease; CKD, chronic kidney disease; CVA, cerebral vascular accident/ DVT, deep vein thrombosis; DM, diabetes mellitus; HLD, hyperlipidemia; HTN, hypertension; IQR, interquartile range; IV, intravenous; IVDU, intravenous drug use.

### Clinical Presentation

Symptoms on presentation were similar between both groups (Table 2), but there was increased incidence of shortness of breath among pregnant patients (41% vs 11%; *P* ≤ .001), and decreased incidence of stroke and altered mental status (5.9% vs 21.3%; *P* = .047). A similar proportion of pregnant and nonpregnant patients (11.7% vs 6.7; *P* = .30) presented for noninfectious reasons (ie, in labor, methadone initiation, trauma). Symptom duration before presentation and presence of fevers were similar between both groups, although the incidence of leukocytosis was lower in the pregnant population (35% vs 61%; *P* = .01). Both groups had rapid attainment of blood cultures (<24 hours) and similar rates of screening for hepatitis C (50%–60%). There were higher rates of urine drug screens among pregnancy-associated IE patients (82.4% vs 50.7%; *P* = .001).

**Table 2.** Clinical Presentation

	Nonpregnant Average			Pregnant			*P* Value
	Median or Count	Percentage	IQR/SD	Median or Count	Percentage	IQR/SD	
Symptom	…	…		…	…	…	…
Fevers	48	32.9		16	47.1	…	.163
Chills	20	13.7		5	14.7	…	>.999
SOB	17	11		14	41.2	…	<.001
Myalgias	21	14.4		2	5.9	…	.257
Back pain	14	9.6		2	5.9	…	.740
CP	11	7.5		3	8.8	…	.730
CVA symptoms/AMS	31	21.3		2	5.9	…	.047
Nonsepsis symptoms	10	6.7		4	11.7	…	.301
Symptom duration before presentation	5	…	(2–10)	6	…	(2.2–7.75)	.170
Febrile on admission	66	45.2		13	38.2	…	.566
Leukocytosis/leukopenia on admission	89	61.0		12	35.2	…	.007
Time to first blood cultures	…	…		…	…	…	…
<24 h	140	95.9		32	94.2	…	.647
>24 h	6	4.1		2	5.9	…	.647
Screened for hep C	78	53.4		22	64.7	…	.256
AB	60	41.1		18	52.9	…	.250
PCR	23	15.8		9	26.5	…	.144
Screened if not known Hepatitis C	40	47.6		8	72.7	…	.017
Urinary drug screen	74	50.7		28	82.4	…	.001
Duration of bacteremia, d	5.1	…	(2–7)	5.9	…	(3–7.5)	.261
Suspected source	…	…		…	…	…	…
IVDU	113	77.4		25	73.5	…	.655
Duration of antimicrobial treatment, wk	6.1	…	(6–6)	5.8	…	(6–6)	.691
Successful completion of antibiotic therapy	69	47.3		20	58.8	…	.256
Surgical indication	100	68.5		25	73.5	…	.681
TEE performed	102	69.8		17	50.0	…	.043

Abbreviations: AB, antibody; AMS, altered mental status; CP, chest pain; CVA, cerebral vascular accident; IQR, interquartile range; IVDU, intravenous drug use; PCR, polymerase chain reaction; SOB, shortness of breath; TEE, transesophageal echocardiogram.

### Endocarditis Details

Approximately 50% of all IE cases across groups affected the tricuspid valve, and the incidence of right- and left-sided lesions was similar in both cohorts. Rates of prosthetic valve endocarditis were similar across groups. Fewer transesophageal echocardiograms were performed on pregnant patients as compared with nonpregnant patients (50% vs 69.8%; *P* = .043).

### Microbiologic Data

Methicillin-susceptible and methicillin-resistant *Staphylococcus aureus* were the most common pathogens in all patients. However, there was greater microbiological variation in nonpregnant patients (Supplementary Figure 2). Duration of bacteremia was similar between both groups, with a median duration of 5 days.

### Maternal Outcomes

There were similar rates of 1- and 2-year mortality between the 2 groups (Table 3). In-hospital mortality was 2.9% in pregnant patients compared with 9.6% in nonpregnant patients (*P* = .31). There were similarly high rates of surgical indications in pregnant vs nonpregnant patients. However, no surgeries were performed during pregnancy; all operations performed in that group occurred postpartum. Total rates of valve replacement (32.4% vs 29.5%; *P* = .84) and valve repair (5.5% vs 5.9%; *P* > .99) were similar between pregnant and nonpregnant groups. When adjusting for the total number of patients meeting surgical indications, rates of intervention remained similar between pregnant and nonpregnant patients (52% vs 51%; *P ≥* .99). With regards to morbidity, there were higher rates of pulmonary embolus (causing occlusion of pulmonary arteries) in pregnancy-related IE, although this did not reach statistical significance (17.6% vs 7.5%; *P* = .098). Pregnant patients had had higher rates of persistent infection despite appropriate antibiotic treatment, although this was also not statistically significant (44.1% vs 28.8%; *P* = .1). We observed similar rates of renal failure, cardiac arrest, acute respiratory distress syndrome, heart failure, arrhythmia, and ICU admission among both groups. For those who underwent surgical replacement or repair, total cardiopulmonary bypass time and time from admission to surgical intervention did not differ between groups. Additionally, rates of postsurgical complications of temporary pacing wires, defibrillator placement, and intracranial hemorrhage were similar between both groups. There were high rates of 30-day readmission (>20%) and relapsed intravenous drug use (IVDU) in both groups (45.2% in the pregnant patients and 51.7% in nonpregnant patients; *P* = .99). Of note, when excluding any cardiac implantable electronic device (CIED)–related IE (n = 2 in the nonpregnant patients) from the statistical analysis, there were no differences in maternal outcomes. Additional maternal outcomes are listed in Supplementary Table 3.

**Table 3.** Maternal Outcomes

	Nonpregnant Patients			Pregnant Patients			*P* Value
	Median or Count	Percentage	IQR/SD	Median or Count	Percentage	IQR/SD	
In-hospital Mortality	14	9.6		1	2.9	…	.310
90-d mortality	14	9.6		1	2.9	…	.310
1-y mortality	22	15.1		5	14.7	…	>.999
2-y mortality	26	17.8		7	20.6	…	>.999
Morbidity	…	…		…	…	…	…
Splenomegaly	30	20.6		4	11.8	…	.332
ARDS	41	28.1		7	20.6	…	.519
Heart failure	41	28.1		9	26.5	…	>.999
Arrhythmia (Afib/SVT/VT)	14	9.6		6	17.6	…	.222
Renal failure	20	13.7		3	8.8	…	.576
Cardiac arrest	7	4.8		1	2.9	…	>.999
ICU admission	102	69.8		21	61.8	…	.414
ICU length of stay, d	5	…	(2–13)	2.5	…	(0–7.75)	.871
Time from admit to surgery consult, d	1	…	(1–3)	5	…	(1–7)	.189
Addiction medicine consult	48	32.9		17	50.0	…	.075
Opiate maintenance therapy started after diagnosis	54	37.0		23	67.6	…	.002
Methadone	24	16.4		14	41.2	…	.220
Suboxone	30	20.6		9	26.5	…	.220
Interventione	…	…		…	…	…	…
Intubation (excluding surgery-associated)	30	20.6		12	35.3	…	.075
Valve replacement	43	29.5		11	32.4	…	.836
AVR	7	4.8		0	0.0	…	.322
MVR	5	3.4		2	5.9	…	.621
TVR	9	6.2		3	8.8	…	.693
Valve repair	8	5.5		2	5.9	…	>.999
Annuloplasty	1	0.7		1	2.9	…	.343
Other	22	15.1		5	14.7	…	>.999
VATS	5	22.7		0	0.0	…	.547
Tracheostomy	7	31.8		0	0.0	…	.283
CABG	3	13.6		0	0.0	…	>.999
Aortic root replacement	4	18.2		2	40.0	…	.303
Other	7	31.8		3	60.0	…	.326
Surgery details	…	…		…	…	…	…
Cardiopulm bypass duration, min	100	…	(72–139)	91	…	(68–103)	.106
Time from admission to surgical intervention, d	13	…	(8–16)	15	…	(10–21)	.192
Length of stay, d	21	…	(11–36)	30.5	…	(16.3–45)	.118
Left AMA	25	17.1		8	23.5	…	.460
30-d readmission	38	26.0		7	20.6	…	.661
Recurrent endocarditis	29	20.0		8	23.5	…	.641
Repeat cardiac surgery	10	6.9		1	2.9	…	.693
Relapsed IVDU	61	51.7		14	45.2	…	.999

Abbreviations: Afib/SVT/VT, atrial fibrillation/supraventricular tachycardia/ventricular tachycardia; AMA, against medical advice; ARDS, acute respiratory distress syndrome; AVR, aortic valve replacement; CABG, coronary artery bypass graft; ICU, intensive care unit; IQR, interquartile range; IVDU, intravenous drug use; MVR, mitral valve replacement; TVR, tricuspid valve replacement; VATS, video-assisted thoracoscopic surgery.

### Delivery and Neonatal Outcomes

Delivery and neonatal outcomes are detailed in Table 4. Of available delivery outcomes, there were 26 (76.4%) live births, 3 (9%) terminations, 4 (5.4%) spontaneous abortions, and 1 intrauterine fetal demise (2.9%). Perinatal mortality (including intrauterine fetal demise and previable delivery) was high at 19.2%. There was a very high preterm delivery rate of 50% (n = 11) with an average live gestational age at birth of 35.0 weeks. Accordingly, the majority of infants (68%) required neonatal intensive care unit admission.

**Table 4.** Delivery and Fetal Outcomes

Delivery and Fetal Outcomes	Total (n = 34)	Percentage
Intrapartum IE	29	85.3
Postpartum IE	5	14.7
Live births	26	70
Perinatal mortality	5	19.2
Termination	3	9
Mode of delivery	…	…
C-section	9	41
Spontaneous vaginal delivery	19	86
C-section indication for maternal instability	3	14
Induction of labor	6	27.3
Average gestational age of live birth, wk	35	…
Preterm delivery	11	50
NICU stay	15	68

Abbreviations: IE, infective endocarditis; NICU, neonatal intensive care unit.

## DISCUSSION

The incidence of IE has increased dramatically over the last decade. In particular, among patients with opioid use disorder, the rates of IE have increased nearly 10-fold in the last decade, from 3.7 per 1 million people per day in 2011 to 30.1 in 2022 [10]. This epidemic also affects women of childbearing age, and as such the rates of IE in pregnancy are increasing [1]. There have been conflicting data regarding overall morbidity and mortality in this population. Our study evaluated a cohort of pregnant and postpartum patients with IE and compared outcomes with a nonpregnant cohort and found mortality and morbidity to be equivalent, yet unacceptably high in both groups. We observed similar 1-year mortality rates (∼15%) in both groups, as well as similar rates of complications such as arrhythmias, cardiac arrest, acute respiratory distress syndrome, and renal failure. These findings are consistent with a recent analysis of the National Readmissions Database that demonstrated that pregnancy did not confer an increased risk of IE-associated mortality or increased adverse outcomes [11]. These combined results support an aggressive approach to treating this population in an attempt to reduce such an unacceptably high mortality rate. While pregnant patients with IE have been considered extremely high risk, in our study postpartum surgical intervention was safe and effective.

We did not observe significant differences in rates of valve repair or replacement between the 2 groups. Although we observed similar rates of surgical intervention (30%) in both groups, all surgeries in the pregnancy-associated IE patients were performed after delivery. Additionally, all patients with an antenatal diagnosis of IE were admitted until delivery and for treatment and coordination of postpartum care. The absence of any statistically significant difference in outcomes or surgical complications suggests that cardiac surgery when indicated can be effectively and safely pursued in the postpartum period. Additionally, the introduction of a multidisciplinary endocarditis team at our institution in 2020 may have improved rates of intervention among pregnant patients.

While rates of mortality were similar between pregnant and nonpregnant patients, the 1-year mortality rate of 15% is still unacceptably high in this young population. A secondary goal of this study was to identify factors related to poor outcomes and identify opportunities for intervention. In this study, we observed high rates (>80%) of IVDU in both groups. The American College of Obstetrics and Gynecology (ACOG) recommends using a questionnaire to screen all pregnant women for substance use disorders. Despite these recommendations, there are existing data that pregnant women presenting to the emergency department are less likely to be tested for alcohol or drug use than nonpregnant patients [12]. Interestingly, in our study we observed much higher—although suboptimal—rates of urinary drug screening in pregnant patients (82.4% and 50.7%, respectively). Similarly, screening rates for hepatitis C were low in our study; only 47.6% of the nonpregnant and 72.7% of the pregnant patients without preexisting hepatitis C received antibody or polymerase chain reaction testing. Overall, our data suggest that there is a need to improve screening for substance use disorders and their downstream complications [13].

Despite increased rates of opioid use disorder screening and availability of treatment, the data suggest that large percentages of hospitalized pregnant patients do not receive medication for opioid use disorder [13, 14]. Historically, patients with injection drug use and/or pregnancy have high rates of patient-directed discharges and failure of antibiotic completion, adding to the increased risk for this vulnerable population [4]. In our study, although substance use disorder was underrecognized and undertreated, in general we did observe higher rates of consultation for addiction medicine and higher rates of discharge on opiate replacement therapy for pregnant patients (statistically significant in pregnant patients). This may be reflective of the medication for opioid use disorder (MOUD) program at the Magee Women's Hospital, which is specifically designed for pregnant patients with substance use disorder and may have increased provider awareness and availability of treatment. It is important to note that this MOUD program was not available outside of pregnancy for the duration of this study, and thus certain treatment opportunities were not necessarily available to nonpregnant patients. Use of additional medicine consultation can lower readmission rates [15], which offers an opportunity for interventions such as increased OUD screening and increased implementation of OUD treatment for all patients.

High rates of concurrent mental health disorders have also been observed in patients with IVDU [16]. Ensuring timely, accurate, and consistent diagnosis of mental health disorders and providing options for treatment represent additional opportunities for intervention.

Our study has highlighted several possible avenues of intervention that could impact the diagnosis and treatment of this vulnerable patient population, including improved diagnosis and treatment of OUD and mental health disorders. In addition, the presence of *S. aureus* bacteremia in pregnancy could raise provider suspicion for IE, which may help lead to earlier diagnosis and treatment. Timely diagnosis of IE in pregnancy allows for rapid implementation of multidisciplinary teams including maternal fetal medicine and cardiology, as these patients require close monitoring, frequent testing, and titration of supportive medications in the event of hemodynamic compromise.

While our study focused primarily on maternal outcomes in IE, we also recorded data regarding fetal outcomes. We observed high rates of fetal mortality (21.5%) and high rates of preterm delivery (46%), which is in line with other published data [11]. High rates of preterm delivery can lead to further neonatal complications including ICU admissions, prolonged lengths of stay, and neonatal abstinence syndrome, which have all been associated with worse neurodevelopmental outcomes and higher disability rates in children [17].

There are many strengths of this study. As mentioned, the previous literature describing maternal outcomes in IE has largely been limited to case reports, with the exception of a recent analysis of the National Readmissions Database assessing adverse outcomes of IE in pregnancy. It is notable that the outcomes examined in the prior study were mortality, thromboembolic events, and ventilation and were largely limited by the database itself. Given that our study involved primary chart review, we were able to include far more detailed analysis of hospital course, complications, and presentation. There are also several limitations of this study worth noting, particularly its retrospective nature and that it was performed at a single site. Given the relatively rare incidence of IE in pregnancy, the total sample size of pregnancy-associated IE is quite small at 34 patients, which limits the power of our statistical analysis and the ability to detect differences between groups. Additional limitations include the generalizability of this study, especially given the homogenous nature of the population and the lack of underrepresented minorities. In addition, the majority of the patients included in this study were diagnosed with endocarditis likely related to IVDU, and this limits generalizability to non-IVDU populations.

This study represents the largest known single-center retrospective review of IE in pregnancy and suggests that pregnancy does not portend an increased risk of mortality and morbidity in this population. The reported data enrich the sparse literature surrounding a vulnerable population of patients and contribute to an understanding of how to improve diagnosis and management of IE in pregnancy.

## Supplementary Material

ofad470_Supplementary_Data

## Supplementary Data

Supplementary materials are available at *Open Forum Infectious Diseases* online. Consisting of data provided by the authors to benefit the reader, the posted materials are not copyedited and are the sole responsibility of the authors, so questions or comments should be addressed to the corresponding author.
