# Supplementary material for: Outcomes of Endocarditis in Pregnancy: A Single-Center Experience
Source: Open Forum Infect Dis. 2023 Sep 20;10(9):ofad470. doi: 10.1093/ofid/ofad470 (PMC10538254; doi:10.1093/ofid/ofad470)
Supplement: ofad470_Supplementary_Data [file ofad470_supplementary_data.docx]

**Supplemental Table 1:**

| **ICD 9:** |
| --- |
| 421.0 Acute and subacute infective endocarditis |
| 421.1 |
| 421.9 |
| 424.90 Endocarditis, valve unspecified |
| 424.99 Endocarditis valve unspecified |
| 424.91 |
| 115.04 |
| 115.14 |
| 115.94 Acute and subacute infective endocarditis in diseases classified elsewhere |
| 112.81 |
| 036.42 |
| 098.84 |
| **ICD 10:** |
| I38 Endocarditis valve unspecified |
| I38.X |
| I33 |
| I33.0 Acute and subacute infective endocarditis |
| I33.9 Acute and subacute infective endocarditis unspecified |
| I39 Endocarditis and heart valve disorders in diseases classified elsewhere (I390) |
| I39.8 Endocarditis and heart valve disorders in diseases classified elsewhere (I390) |
| I01.1 |
| **Transthoracic Echocardiography and Transesophageal Echocardiography Procedure Codes (CPT) codes** |
| C8929 |
| C8930 |
| 93303 |
| 93304 |
| 93306 |
| 93307 |
| 93308 |
| 93320 |
| 93321 |
| 93325 |
| 93350 |
| 93312 |
| 93313 |
| 93314 |
| 92215 |
| 93316 |
| 93317 |
| 93318 |
| 93355 |

**Supplemental Figure 1:** Cohort development of Pregnant and non-Pregnant patients with Infective Endocarditis


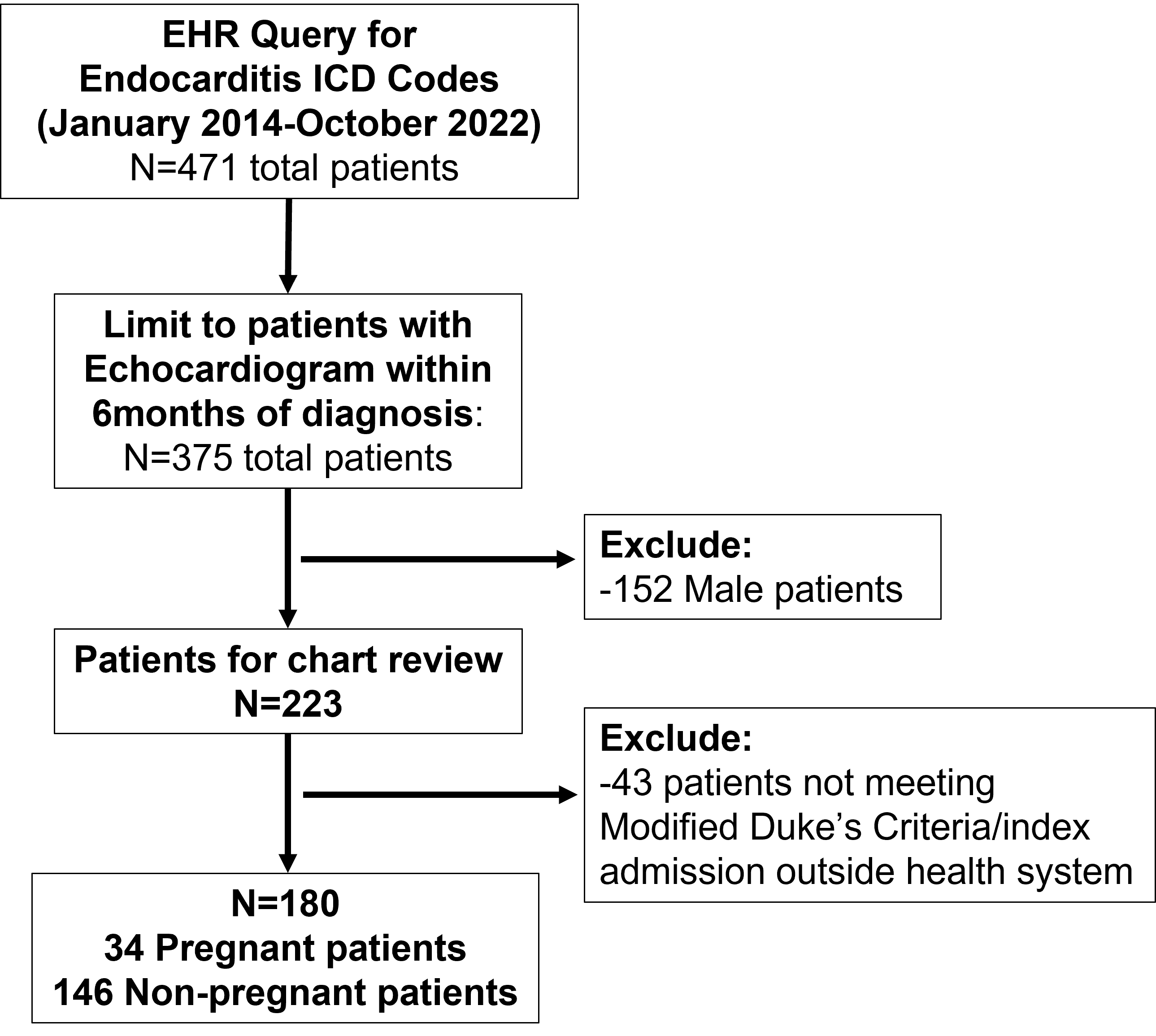


**Supplemental Table 2.**

| **Maternal outcomes** |
| --- |
| In-hospital Mortality |
| 90-day mortality |
| 1-year mortality |
| 2-year mortality |
| Embolic phenomenon |
| Stroke |
| Pulmonary embolus |
| Pulmonic septic emboli |
| Other septic emboli (arthritis, renal infarct etc) |
| Splenomegaly |
| Heart failure |
| Acute respiratory distress syndrome |
| Renal failure |
| Arrhythmia (Atrial fibrillation, SVT, VT/VF) |
| Cardiac Arrest |
| Valvular details |
| Regurgitation severity |
| Ejection Fraction on TTE/TEE |
| ICU admission |
| ICU length of stay (days) |
| Time from admit to Cardio-Thoracic surg consult (days) |
| Addiction medicine consult |
| Initiation of opiate maintenance therapy while inpatient |
| **Intervention:** |
| Intubation |
| RHC/LHC |
| Inta-aortic balloon pump |
| Impella |
| ECMO |
| Surgical Indications |
| Heart failure from valve dysfunction |
| Persistent bacteremia or sepsis (5-7 days) |
| Abscess, heart block or other destructive penetrating lesion |
| Recurrent emboli despite appropriate antibiotic therapy |
| Large Vegetation (greater than 1.0 - 1.5 cm) |
| Surgical intervention |
| Valve replacement |
| Valve repair |
| Annuloplasty |
| Other |
| Surgical intervention details |
| Cardio-pulmonary bypass duration (mins) |
| Time from admission to surgical intervention |
| Temporary pacing wire placement |
| PPM placement |
| ICD placement |
| Intracranial hemorrhage post-operatively |
| Length of Stay |
| 30 day readmission |
| **Pregnancy Outcome: Obstetric complication** |
| Hypertensive Disorder of Pregnancy |
| Postpartum hemorrhage |
| Mode of delivery |
| Spontaneous vaginal delivery |
| Ceasarean section |
| Indication for C-section |
| Induction of labor |
| Intrauterine fetal demise |
| Spontaneous abortion (SAB) |
| Elective termination (TAB) |
| Gestational age at birth/termination |

**Supplemental Table 3: Endocarditis details and Maternal outcomes**

|  | **Non-Pregnant** | | | **Pregnant** | | | **P-Value** |
| --- | --- | --- | --- | --- | --- | --- | --- |
|  | Median or Count | Percentage | IQR/ SD | Median or count | Percentage | IQR/ SD | p-value |
| **Valve involvement** |  |  |  |  |  |  |  |
| **Aortic** | 26 | 17.8 |  | 5 | 14.7 |  | 0.804 |
| **Mitral** | 42 | 28.8 |  | 12 | 35.3 |  | 0.534 |
| **Pulmonic** | 3 | 2.1 |  | 2 | 5.9 |  | 0.239 |
| **Tricuspid** | 76 | 52.1 |  | 16 | 47.1 |  | 0.704 |
| **No valve** | 14 | 9.6 |  | 5 | 14.7 |  | 0.364 |
| **Type of valve** |  |  |  |  |  |  |  |
| **Native** | 128 | 87.7 |  | 28 | 82.4 |  | 0.408 |
| **Prosthetic** | 19 | 13.0 |  | 2 | 5.9 |  | 0.375 |
| **Valvular details** |  |  |  |  |  |  |  |
| **Regurgitation severity** |  |  |  |  |  |  |  |
| **Mild regurgitation** | 37 | 25.3 |  | 9 | 26.5 |  | 0.681 |
| **Mod regurgitation** | 33 | 22.6 |  | 9 | 26.5 |  | 0.655 |
| **Severe regurgitation** | 56 | 38.4 |  | 10 | 29.4 |  | 0.430 |
| **No regurgitation** | 20 | 13.7 |  | 6 | 17.6 |  | 0.590 |
| **EF on TTE/TEE (%)** | 57.6 |  | (55,60) | 56.3 |  | (55,57.5) | 0.313 |
| **Vegetation size** |  |  |  |  |  |  |  |
| **1-dimension** | 1.8 |  | (1.1,2.4) | 1.9 |  | (1.0,2.4) | 0.863 |
| **2-dimension** | 1.0 |  | (0.58,1.4) | 1.0 |  | (0.63,1.55) | 0.329 |
| **>1 vegetation** | 4 | 2.7 |  | 5 | 14.7 |  | 0.013 |
| **No veg** | 21 | 14.4 |  | 6 | 17.6 |  | 0.601 |
| **Time from admission to TEE** | 3 |  | (2,4) | 3 |  | (1,10) | 0.529 |

|  | **Non-Pregnant** | | | **Pregnant** | | | **P-value** |
| --- | --- | --- | --- | --- | --- | --- | --- |
|  | Median or Count | Percentage | IQR/SD | Median or count | Percentage | IQR/SD | P-value |
| **Persistent bacteremia or sepsis (5-7 days)** | 42 | 28.8 |  | 15 | 44.1 |  | 0.102 |
| **Abscess, heart block or other destructive penetrating lesion** | 24 | 16.4 |  | 3 | 8.8 |  | 0.423 |
| **Recurrent emboli despite appropriate antibiotic therapy** | 10 | 6.9 |  | 11 | 32.4 |  | >0.001 |
| **Large Vegetation (greater than 1.0 - 1.5 cm)** | 72 | 49.3 |  | 18 | 52.9 |  | 0.849 |
|  |  |  |  |  |  |  |  |
| **Embolic phenomenon** |  |  |  |  |  |  |  |
| **CVA** | 30 | 20.6 |  | 9 | 26.5 |  | 0.490 |
| **PE** | 11 | 7.5 |  | 6 | 17.6 |  | 0.098 |
| **Pulmonic septic emboli** | 91 | 62.3 |  | 22 | 64.7 |  | 0.846 |
| **Other septic emboli** | 76 | 52.1 |  | 12 | 35.3 |  | 0.089 |
| **Splenic/ hepatic infarct** | 15 | 10.3 |  | 4 | 11.8 |  | 0.761 |
| **Renal infarct** | 12 | 8.2 |  | 1 | 2.9 |  | 0.467 |
| **Septic arthritis** | 27 | 18.5 |  | 4 | 11.8 |  | 0.454 |
| **Empyema** | 14 | 9.6 |  | 1 | 2.9 |  | 0.310 |
| **Muscle abscess** | 11 | 7.5 |  | 2 | 5.9 |  | >0.999 |
| **Spinal abscess** | 17 | 11.6 |  | 1 | 2.9 |  | 0.203 |
| **Other** | 6 | 4.1 |  | 0 | 0.0 |  | 0.596 |
|  |  |  |  |  |  |  |  |
| **Complications** |  |  |  |  |  |  |  |
| **RHC/LHC** | 8 | 8.0 |  | 0 | 0.000 |  | 0.144 |
| **ECMO** | 3 | 3.0 |  | 0 | 0.000 |  | >0.999 |
| **Surgical Indication and no intervention** | 51 | 51 |  | 13 | 52 |  | >0.999 |
| **Temp pacing wire** | 37 | 25.3 |  | 10 | 29.4 |  | 0.666 |
| **PPM placement** | 16 | 11.0 |  | 2 | 5.9 |  | 0.532 |
| **Intracranial hemorrhage post-operatively** | 1 | 0.7 |  | 1 | 2.9 |  | 0.343 |

Supplemental Figure 2a. Infectious Agent- Non-Pregnant patients

Supplemental Figure 2a. Infectious Agent- Pregnant patients

Supplemental Figure 2c. Antibiotics- Non-Pregnant patients

Supplemental Figure 2d. Antibiotics- Pregnant Patients
